# LIFESTYLE CHANGES AFTER FRAILTY ONSET AMONG MIDDLE-AGED AND OLDER ADULTS IN THE UNITED STATES

**DOI:** 10.1093/geroni/igad104.3042

**Published:** 2023-12-21

**Authors:** Hui-wen Xu, Yu-Ming Chen, Zhou Yang, Bei-bei Xu

**Affiliations:** Peking University, Beijing, Beijing, China (People’s Republic); Peking University, Beijing, Beijing, China (People’s Republic); Peking University, Beijing, Beijing, China (People’s Republic); Peking University, Beijing, Beijing, China (People’s Republic)

## Abstract

Frailty could be the teachable moment to promote a lifestyle change for middle-aged and older adults. This study aimed to investigate the impact of sociodemographic characteristics on lifestyle changes among frail adults aged ≥50 years. A total of 5,440 frail individuals aged ≥50 years from the Health and Retirement Study (HRS) 2004-2020 were included. The Paulson–Lichtenberg Frailty Index was used to measure frailty. Lifestyle included smoking status, alcohol consumption, physical activity, and sleep problems. Logistic regression models were used to examine the associations between sociodemographic characteristics and lifestyle change among frail participants. After frailty, a higher proportion of individuals reported maintaining their smoking and drinking status without change. Compared to increased physical activity, more people reported reducing their low, moderate, and high-intensity physical activity. Similarly, a greater proportion of individuals reported worsened sleep problems compared to those who experienced improvements in sleep problems. Age was negatively associated with quitting smoking and increased light, moderate, as well as vigorous physical activity, while was positively associated with improving sleep problems. Males were more likely to quit smoking, quit drinking, and increase light physical activity. Individuals with medium and high education levels were more likely to quit drinking and less likely to increase moderate physical activity. A sensitivity analysis based on the 4-year interval between pre-diagnosis and post-diagnosis of frailty showed that being unmarried was associated with an increased likelihood of quitting smoking. Some people reported lifestyle changes after frailty. Age, gender, marital status, and education levels may affect lifestyle changes after frailty.

